# Supplementary material for: Prophylactic anticoagulants to prevent venous thromboembolism in patients with nephrotic syndrome—A retrospective observational study
Source: PLoS One. 2021 Jul 28;16(7):e0255009. doi: 10.1371/journal.pone.0255009 (PMC8318234; doi:10.1371/journal.pone.0255009)
Supplement: S1 Table — (PDF) [file pone.0255009.s001.pdf]

S1 Table. Outcome frequency in 77 patients with NS, diabetic nephropathy excluded.

Outcome	*PAC (n =39)*	*No PAC (n=38)*	*p-value*
Venous thromboembolism	4 (10.3)	2 (5.2)	0.675
Major bleeding	2 (5.1)	2 (5.2)	1.000
Minor bleeding	7 (17.9)	6 (15.8)	1.000
Death	2 (5.1)	0	0.494

Results presented as n (%). Comparison between 77 patients with NS, 18 patients with diabetic nephropathy excluded, comparison of PAC- and no PAC-group was made using Fisher's exact test.
